# Severe cerebral abscess associated with pulmonary arteriovenous fistula: case report and literature review

**DOI:** 10.1186/s41016-018-0137-4

**Published:** 2018-11-05

**Authors:** Chunzhao Li, Shuyu Hao, Jiangfei Wang, Zhixian Gao, Nan Ji

**Affiliations:** 1 Beijing Tiantan Hospital, Capital Medical University, Beijing, China; 2 Beijing Key Laboratory of Brian Tumor, Beijing, China; 3 China National Clinical Research Center for Neurological Diseases Beijing, Beijing, China

**Keywords:** Brain abscess, Hyperhemoglobinemia, Hypoxemia, Pulmonary arteriovenous fistula

## Abstract

**Background:**

A rare case of cerebral abscess concurrent with pulmonary arteriovenous fistula (PAVF), hyperhemoglobinemia, and hypoxemia was reported.

**Case presentation:**

A 37-year-old man was admitted with a headache, nausea, vomiting, fever, and numbness of the left cheek and upper limb for 10 days. Cerebral magnetic resonance imaging (MRI) shows the lesion in his right frontal lobe. Blood gas analysis indicated lower blood oxygen saturation level, and blood routine test showed hemoglobin elevation. Craniotomy for the lesion and decompressive craniotomy were performed. Brain abscess was confirmed by pathology examination. The chest computed tomography angiography (CTA) revealed a pulmonary arteriovenous fistula (PAVF) in his right lower lung. After 1 month, embolization of PAVF was performed. Anoxic symptom improved after surgery. Cranioplasty was performed after 7 months.

**Conclusion:**

The author reported a rare case of cerebral abscess associated with pulmonary arteriovenous fistula. Brain abscess, hyperhemoglobinemia, and hypoxemia might be secondary to PAVF. Treatment of patients includes not only craniotomy for abscess removal but also embolization of PAVF which can prevent recurrence of brain abscess.

## Background

Cerebral abscess is the infection of the cerebral parenchyma. It is mainly caused by contiguous infections from adjacent or distant infections transferred hematogenously as well as from neurosurgery and injury. The causative pathogen of cerebral abscess is associated with period, geographic distribution, age, underlying medical and/or surgical conditions, and mode of infection [1]. Although all of these are potential routes, there are still 20–30% of cases without an identified source of infection [2]. Nowadays, one reason of a no-source distant infection has been known as cardiac right to left shunt disease [3]. As the pulmonary arteriovenous fistula (PAVF) has directed a connection between the artery and the vein, the blood flows without going through the capillary bed [4], so it also lacks oxygen exchanging [5]. Due to the blood-brain barrier and the abundant blood supply, the brain abscess caused by PAVF is only 5% [6].

Here, the authors reported a rare case of cerebral abscess caused by idiopathic PAVF with hyperhemoglobinemia and hypoxemia.

## Case presentation

**Fig. 1:**
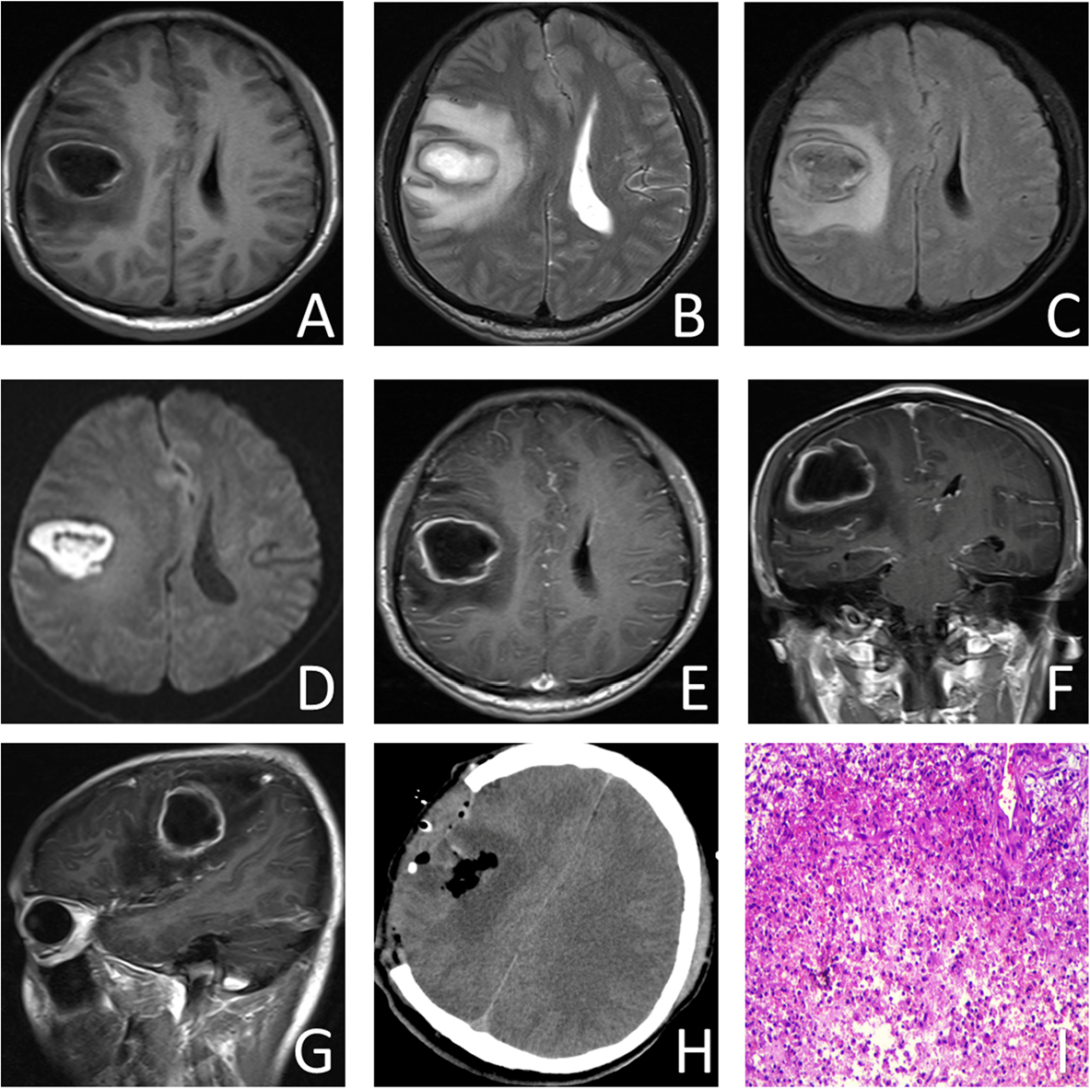
**a**–**g** Brain magnetic resonance imaging showed a 33 × 41 × 40 mm ring-enhanced capsule in the right frontal lobe with severe perilesional edema which leads to midline shifting to the left side, and the right ventricle was metamorphose. Diffusion weighted MR of the enhanced area revealed the lesion is an abscess. **h** Postoperative CT scan showed regular changes of post right temporoparietal craniotomy and deficiency of the right temporoparietal bone. **i** Pathological manifestation of the mass (HE× 100)

**Fig. 2:**
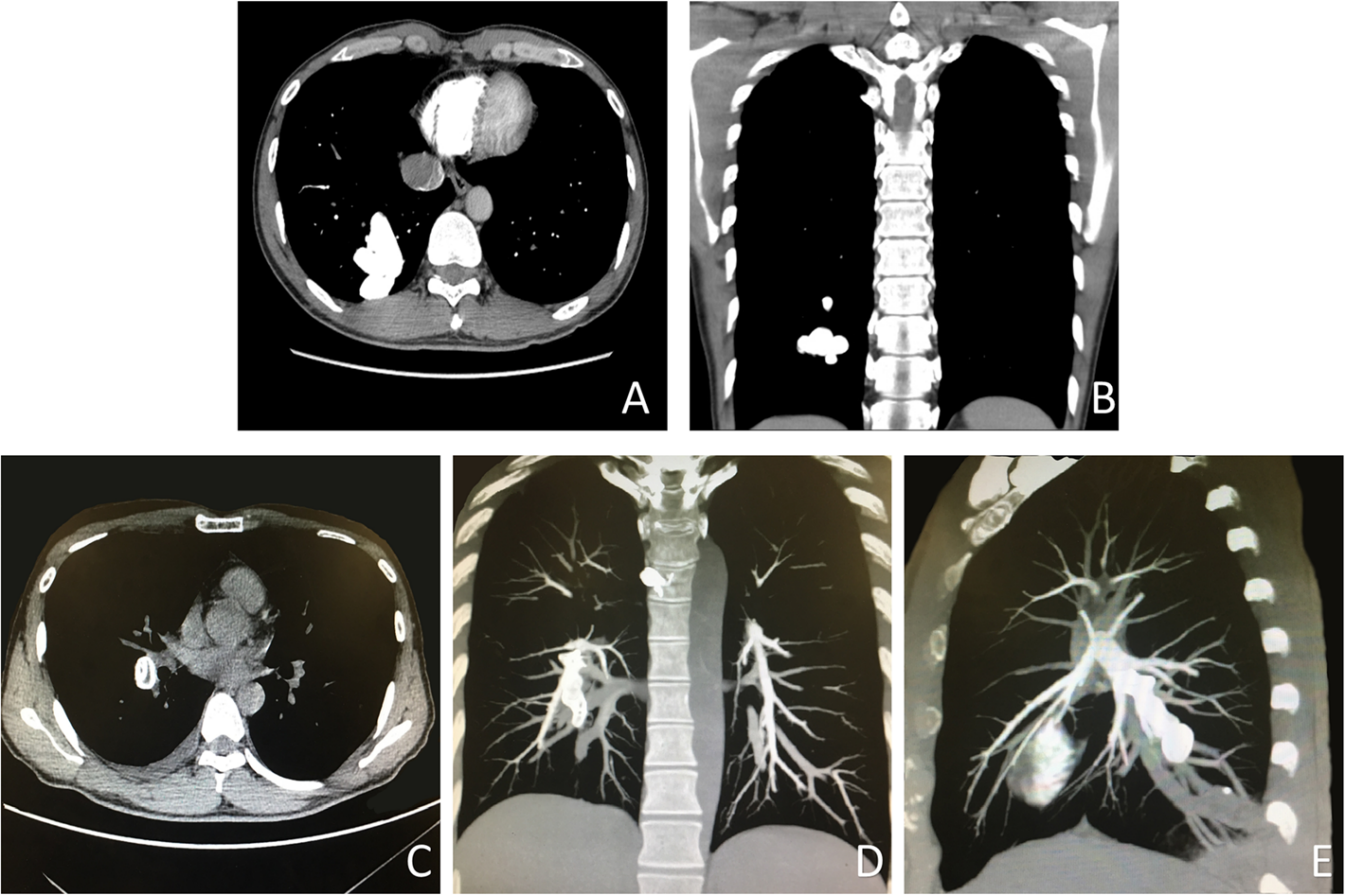
**a**–**b** Preoperative chest computed tomography angiography showed a group of tortuous vascular, considered as fistulous vascular abnormality on the right lower lung field. **c**–**e** Postoperative CTA: Inferior lobe of the right lung and posterior basal segmental of pulmonary artery have high density of implant. No other pulmonary arteries have filling defect

A 37-year-old man was admitted by emergency with a headache, nausea, vomiting, fever, numbness of the left cheek and upper limb for 10 days which had aggravated in 1 week. Physical examination found sanity, poor mental state, drowsiness, cyanosis, clubbing, low appetite, nuchal rigidity, shallowing left nasolabial fold, and loll left and left limb muscle force at grade IV, with no other cranial nerves deficit. No special medical history was found. No other infection was found. Preoperative blood bacteria culture proved negative. Hemoglobin (HGB) was 213 g/L, and RBC was 6.89 × 10^12^/L. Arterial blood gas analysis revealed a pH of 7.41, PCO_2_ is 33 mmHg, PO_2_ is 66 mmHg, oxygen saturation is 93%, and HCO_3_^−^ is 20.9 mmol/L on room air. Computed tomography (CT) scan of the brain indicated a low-density shadow in the right frontal lobe with the edge enhancing. Brain MRI showed a 33 mm × 41 mm × 40 mm ring-enhanced capsule in the right frontal lobe with severe perilesional edema which leads to the midline shifting to the left side, and the right ventricle was metamorphose (Fig. 1). Chest x-ray revealed a high-density nodule with an irregular shape in the right lower lung. To make further diagnosis, pulmonary artery computed tomography angiography (CTA) which is a diagnostic criterion of pulmonary AVF was performed to confirm AVF (Fig. 2a, b). To avoid brain hernia for high intracranial pressure, craniotomy for the lesion and decompressive craniotomy were performed. During the operation, the lesion capsuled by a green wall was totally resected and there was a deep yellow abscess inside. The analysis on germiculture and sensitive test of the surgically removed brain abscess showed that it was microaerophilic streptococcus infected. After the operation, we used intravenous vancomycin hydrochloride (1000 mg q12h) according to the drug susceptibility test result. We suggested him to have an antibiotic therapy for 4 weeks and therapeutic embolization of PAVF. One month later, embolization using Amplatzer vascular plugs was performed on him (Fig. 2c–e). The postoperative symptoms of the patient were obviously improved. According to the Curacao criteria, which is the diagnosis of hereditary hemorrhagic telangiectasia, [7] (Table 1), the patient’s family history and symptoms accord with one criteria that suggest underlying HHT. After embolization, the patient’s blood oxygen saturation and hemoglobin returned to normal.

**Table 1:** Curacao criteria for the diagnosis of HHT

1. Epistaxis—spontaneous, recurrent nosebleeds	
2. Telangiectases—multiple, at characteristic sites (lips, oral cavity, fingers, nose)	
3. Visceral lesions—such as gastrointestinal telangiectasia (with or without bleeding), pulmonary arteriovenous fistula (AVF), hepatic AVF, cerebral AVF, spinal AVF	
4. Family history—a first-degree relative with HHT	

The third criterion indicates a definite diagnosis of HHT

The second criterion indicates a possible or suspected HHT

## Discussion

Pulmonary arteriovenous fistula is known as an abnormal vascular connection between the artery and the vein. The fistula has always been an intervening thin-wall aneurysmal sac consisting of one layer of endothelial cells and is easy to fracture leading to hemoptysis [8]. Kjeldsen has reported that gastrointestinal bleeding and history of untreated PAVFs causing respiratory symptoms contributing to the increasing mortality of the Danish population, especially below the age of 60 years [9]. Most patients are congenital or developmental in origin; however, PAVF can be followed by chest surgery, postoperative congenital heart disorders, chest trauma, cirrhosis, etc. [10].

The most prominent complication of PAVF is neurologic diseases which have been observed at nearly 35% (range, 5–56%) [11], and the incidence of brain abscess is approximately 5%. So, the neurosurgeon can meet the PAVF patients whose initial symptom is brain abscesses [11], hemiplegia [12], seizures, or migraines [13], but the symptom of PAVF, such as dyspnea, hemoptysis, cyanosis, or asymptomatic, may be covered [14]. In addition, PAVF can lead to various neurological complications such as stroke (20%), paradoxical embolism [15], and other systematic diseases such as dyspnea (50%), migraines (30%) [13], hemoptysis (15%), epistaxis (73%) [16], cerebral arteriovenous fistula (5%), porencephaly (5%), encephalomalacia (5%), and atrophy (9%) [17]. As for physical signs, cyanosis (29%), clubbing (19%), and auscultation murmur (34%) can be found. This patient did not have chest pain, hemoptysis, cough, palpation, or epistaxis but has high hemoglobin and cyanosis which is also an indication of long-time anoxia.

Chest x-ray is a basic examination for every patient, in which the lesion appeared as oval or round. Furthermore, pulmonary CT is clearer and shows the structure of vascular system. However, the gold standard of diagnosing is pulmonary angiography as well as other chemical examinations such as blood test and blood gas analysis. The patient was considered as having pulmonary disease when the examination showed his high HGB, polycythemia, abnormal blood gas analysis, and chest x-ray as well as his physical signs especially cyanosis. After pulmonary angiography, PAVF was diagnosed.

According to the recent British Thoracic Society Clinical Statement on PAVFs, it is recommended that all patients with radiologically visible PAVFS need embolization irrespective of size. Since the 1980s, embolization has replaced surgery gradually due to being less invasive and easy to repeat. However, surgery cannot be excluded completely as it is used to control hemorrhage in an emergency procedure [8] and in any other lesion that cannot be treated by embolization. The patient’s treatment was performed with an embolization device, using Amplatzer vascular plugs which were the most recent devices. Antibiotic prophylaxis is highly recommended by Borrero and Shovlin [18, 19].

## Conclusion

In conclusion, clinicians need to consider pulmonary arteriovenous fistula as a reason of cerebral abscess, and examination of PAVF should depend on patients’ symptoms and signs. Therapy on brain lesion, which is always life-threatening, first was highly recommended, and PAVF must be radically cured as soon as possible; otherwise, cerebral abscess will recrudesce soon or later.

## Abbreviations

CT: Computed tomography
CTA: Computed tomography angiography
HGB: Hemoglobin
HHT: Hereditary hemorrhagic telangiectasia
MRI: Magnetic resonance Imaging
PAVF: Pulmonary arteriovenous fistula
RBC: Red blood cell
